# Isokinetic trunk strength in the teenagers with and without low-back pain: a comparative study

**DOI:** 10.1186/1748-7161-7-S1-O38

**Published:** 2012-01-27

**Authors:** JC Bernard, A Pujol, S Boudokhane, J Deceuninck, E Chaleat-Valayer

**Affiliations:** 1 Croix Rouge française-CMCR des Massues, Lyon, France

## Objectives

To evaluate isokinetic trunk strength in low-back pain (LBP) teenagers, its relations with the clinical measures, and comparison analysis with healthy teenagers.

## Materials and methods

This study has included two groups of 22 LBP and 22 healthy teenagers, aged 11-13 years. We have measured the isokinetic trunk strength in each group on the Cybex trunk extension/flexion machine.

## Results

No significant difference was found between isokinetic peak torque, total work and mean power in extension and flexion in the two groups. Other than, the control group had a higher mean power extension in 120°/s than the LBP group with a nearly significant p. Significant correlations between isokinetics total work at 120° in flexion (r=0,461) and in extension (r=0,475) were observed with the body mass index. A high correlation was also found between the total work at 120° in extension (r=0,668) and the number of hours per week of sport activities. The mean power in extension at 120°/s was correlated positively (r=0,453) to the lumbar radiological lordosis. The endurance of agonist (flexors) and antagonist (extensors) ratio was high in both populations compared to the expected values in general population (0,91 vs 0,89).

## Conclusions

This study reveals no significant difference in the isokinetic trunk strength of both flexors and extensors in the two groups. Based on the results, its interest to study the effect of isokinetic rehabilitation to remove the inhibition at high speed. Isokinetic strength parameters, as measured in this study, do not seem to explain the occurrence of low back pain among children.

